# Adaptor complex-mediated trafficking of Newcastle disease virus fusion protein is regulated by the YLMY motif of its cytoplasmic tail

**DOI:** 10.1080/21505594.2022.2136433

**Published:** 2022-10-18

**Authors:** Yawen Bu, Qingyuan Teng, Delan Feng, Rong Liang, Haoran Wang, Xuehui Zhang, Xiao Li, Wenfeng Jia, Jia Xue, Ye Zhao, Guozhong Zhang

**Affiliations:** Key Laboratory of Animal Epidemiology of the Ministry of Agriculture, College of Veterinary Medicine, China Agricultural University, Beijing, China

**Keywords:** Newcastle disease virus, NDV, F protein, cytoplasmic tail, YLMY motif, AP transport

## Abstract

Previously, we reported that the mediation of Newcastle disease virus (NDV) pathogenicity by the ^524^YLMY^527^ motif depends mainly on the regulation of F protein transport to the cell surface. The virus and host determinants that govern this intracellular trafficking remain unknown. Here, we confirmed that host adaptor protein (AP) complexes are involved in NDV infection using small interfering RNA. The transport of viral F protein to the cell surface depends on host transport proteins. We observed that the trends for host expression of AP complexes AP1M1 and AP2M1 were similar to those of mutated F proteins, especially in the membrane protein. NDV F protein interacted with AP1M1 and AP2M1, and the YLMY motif influenced this interaction. Knockdown of AP1M1 or AP2M1 suppressed the intracellular and extracellular virus titre of mutated-YLMY-motif NDVs, especially rSG10*-F/Y527A and rSG10*-F/Y524AY527A, to varying degrees. Therefore, the YLMY motif regulates AP-mediated viral F protein transportation from the cytoplasm to the cell surface and subsequently affects viral titer. We further found that the YLMY-motif mutants were differently associated with the process of AAK1 and GAK kinase-mediated AP – viral F protein interaction. These data demonstrate that the essential YLMY motif located in the NDV F protein cytoplasmic tail recruits AP to direct the F protein to the cell surface, which is necessary for its ability to affect virus budding. This study provides support for a deeper understanding of virus and host determinants that facilitate virus trafficking, which can be exploited in the design of novel antiviral therapies.

## Introduction

Newcastle disease virus (NDV) is a highly pandemic and widespread pathogen among avian species and generates great economic losses to poultry industries all over the world [1]. NDV, also known as *Avian orthoavulavirus 1*, belongs to the genus *Orthoavulavirus* in the family Paramyxoviridae. It has an approximately 15-kb non-segmented negative-sense single-stranded RNA genome encoding six structural proteins: nucleoprotein (NP), phosphoprotein (P), matrix protein (M), fusion protein (F), haemagglutinin-neuraminidase (HN), and large protein (L) [2,3]. F is a type I protein and mediates invasion of the cellular membrane during the viral cell-entry stage [4]. It is initially synthesized as an inactive precursor, named F_0_, which is then activated by proteolytic cleavage into the disulphide-linked F_1_–F_2_ complex during transport to the Golgi complex [5,6]. The F_1_ subunit, from the *N*-terminus to the C-terminus, sequentially contains the fusion peptide (FP), two hydrophobic heptad repeat (HR) domains (HRA and HRB), and the transmembrane (TM) domain [7]. All these protein structures are extracellular domains, which are known to be critical to viral replication [8], pathogenicity [9], and protein procession [10]. Research conducted on other type I glycoproteins (in retroviruses, lentiviruses, herpesviruses, and other paramyxoviruses) has indicated that the cytoplasmic tail (CT) of NDV F protein also plays a key role in regulating viral entry, cleavage, and fusion [11–14]. However, the specific role of the CT domain is still the least well-understood.

Paramyxovirus surface glycoproteins each contain a short CT, and the CTs of different paramyxovirus do not possess obvious conservation in their amino acid distribution or sequence length. The CT participates in various stages of viral infection. Extensive evidence indicates a vital role of the CT in ectodomain oligomerization and folding [15,16]. Deletion of the CT domain of parainfluenza virus 5 (PIV5) HN protein prevented its assembly into an oligomer and its subsequent transportation to the plasma membrane [17]. Other reports have found that the CT of F protein plays an important role in particle formation because this region is associated with glycoprotein integration into packaged virions and M protein interaction [18–20]. The influence of the glycoprotein CT on virion assembly seems to be a shared characteristic of various RNA viruses, as CT truncations in the glycoprotein of rhabdovirus or in the haemagglutinin and neuraminidase proteins of influenza A virus cause serious defects in virion formation [21,22]. It is supposed that the function of glycoprotein CT is determined by certain functional amino acid sequences or signals rather than simply by a defined length. For Sendai virus (SeV), mutations in the TYTLE motif of the F protein CT had the greatest effect on the gathering and formation of viral components on the cell surface compared with random truncations and mutations [22]. The CTs of Nipah (NiV) and Hendra (HeV) F proteins contain a YXXΦ motif that is required for the internalization of the protein by cells. NiV F proteins expressed from plasmid DNA are located primarily at the basolateral surface of epithelial cells, and this location depends on the YXXΦ motif in the CT of NiV F [23]，[24]. In the CT of human respiratory syncytial virus (HRSV) F protein, a four-amino-acid sequence containing a key phenylalanine residue has been shown to coordinate the assembly and budding of virus filaments [25,26]. In addition, the CTs of glycoproteins of several other viruses contain sequence motifs related to the transport of newly synthesized glycoproteins from the endoplasmic reticulum (ER) to specific locations [27,28]. Furthermore, such tyrosine-based motifs are often associated with endocytosis signals. Reportedly, the replacement of tyrosine, analogous to the substitutions shown to abolish human immunodeficiency virus (HIV) polarized budding in epithelial cells, results in decreased endocytosis and the accumulation of lymphocytes at the surface of cells infected with either HIV-1 or simian immunodeficiency virus (SIV) [29].

Intracellular membrane traffic depends on the interaction between adaptor protein (AP) complexes and their cargo protein. APs are a family of heterotetrameric proteins that coordinate the formation of vesicles and their subsequent transport through different intracellular pathways [30,31]. To date, five different complexes have been identified, and each of them is composed of two large (β and α, γ, δ, or ε) subunits, a medium (μ) subunit, and a small (σ) subunit [32]. AP-1 is involved in the back-and-forth transport of cargo protein within the trans-Golgi network (TGN) and endosomes [30]. AP-2 acts in the endocytic pathway at the plasma membrane and is the most extensively studied AP [31]. AP-3 participates in the transport of specific proteins in the endo-lysosomal pathway. AP-4 has been suggested to contribute to the basolateral protein sorting of polarized cells. AP-5, the most recently discovered AP complex member, has been reported to function in late endosomes [33].

AP complexes recognize plentiful cargo proteins according to typical classification motifs present in the transmembrane protein CTs. Two typical sorting motifs have been described: μ-adaptins, bounded by the tyrosine motif YXXΦ (in which Y = tyrosine, X = any amino acid, and Φ = any bulky hydrophobic amino acid), and σ-adaptins, bounded by the di-leucine motif D/ExxxLL/I (in which D = aspartate, E = glutamate, L = leucine, and I = isoleucine) [34–37]. Some less conserved cargo protein-sorting motifs can also be bound by AP complexes. Cargo protein binding requires large conformational changes in the AP complexes. The µ-adaptin C-terminal domain containing the YXXΦ motif-binding domain is intercalated between large adaptins, while µ1 and µ2 are phosphorylated by two host-cell kinases (AP-2-associated protein kinase [AAK1] and/or cyclin G-associated kinase [GAK]), which supports conformational changes and binding of cargo protein [38–40]. Substantial evidence indicates that many viruses utilize cellular AP complexes as conserved viral foci to allow efficient viral proliferation and evasion of host immune defences. The most well-illustrated cases are the interactions of HIV and human herpesviruses (HHVs) with the AP complexes, the mechanism of which is related to the recognition of specific viral glycoprotein CT motifs by APs that subsequently mediate glycoprotein transport. For HIV and SIV, a conserved GYXXΦ motif located in the CT of gp41 mediates endocytosis by interacting with AP-2 [41].

The NDV F protein CT contains 31 amino acids and is highly conserved among different viral strains. In our previous research, we found a highly conserved YLMY motif in the NDV F protein CT that could regulate viral replication and pathogenicity by affecting F protein transportation to the cell membrane [42]. Here, we identified the host transport protein (AP) that is involved in the NDV lifecycle and found that the YLMY motif regulates the AP-mediated intracellular trafficking of viral F protein. The results indicate that the AP-directed trafficking is mediated by the YLMY motif of NDV F protein and identify a new protein determinant of host – virus interaction, which will be useful for developing therapeutics to treat viral infection.

## Materials and methods

### Cells and viruses

Baby hamster kidney (BHK-21) cells stably expressing T7 RNA polymerase (BSR-T7/5) were grown in Dulbecco’s modified Eagle’s medium (DMEM; Gibco, Grand Island, NY, USA) containing 10% foetal bovine serum (FBS; Gibco). The YLMY-motif mutants were generated from genotype Ⅶd NDV strain SG10 and stored in our laboratory. The mean death time (MDT) and the intracerebral pathogenicity index (ICPI) values of wildtype (rSG10*) and the YLMY-motif mutant viruses, measured in our previous study (MDT = 50–60 h and ICPI is between 1.50 and 1.80), indicate that the parental and YLMY-motif mutant viruses are all virulent strains [37].

### Co-immunoprecipitation (co-IP) and western blot assays

Primers were designed on the basis of the nucleotide sequences of the μ subunits of mouse AP family proteins (AP1M1–AP5M1) downloaded from GenBank in NCBI (primer sequences are listed in Additional file 1: Table S1). The total RNA of BSR-T7/5 cells was extracted and transcribed into cDNA, which was used as the template to amplify AP1M1–AP5M1. Target fragments of AP1M1–AP5M1 were inserted into the vector pCMV-Myc using homologous recombination, generating myc-tagged plasmids named pCMV-Myc-AP1M1, pCMV-Myc-AP2M1, pCMV-Myc-AP3M1, pCMV-Myc-AP4M1, and pCMV-Myc-AP5M1, respectively. The construction of the desired plasmids was verified by sequencing. For Flag-tagged F-gene plasmid construction, NDV strain SG10 was used as a template to amplify the complete F gene with up- and down-stream homologous arms to the pRK5-Flag plasmid. After the construction of pRK5-Flag-F via homologous recombination, plasmids with YLMY-motif point mutations (pRK5-Flag-F/Y524A, pRK5-Flag-F/Y527A, and pRK5-Flag-F/Y524AY527A) were generated by separately introducing the mutation-containing YLMY-motif sequences into pRK5-Flag-F by applying PCR-based site-directed mutagenesis (primer sequences are shown in Additional file 1: Table S2). Cells were separately transfected with each of the plasmids described above, after which the cells were lysed with 500 µl of Pierce® IP Lysis Buffer (Thermo Fisher Scientific, Waltham, MA, USA). The obtained lysates were incubated with Anti-Flag M2 affinity gel overnight at 4 °C (Sigma-Aldrich, St. Louis, MO, USA). For endogenous co-immunoprecipitation (co-IP), cells were separately infected with each of the YLMY-motif-mutant viruses, and then lysates of these cells were added to Protein A/G magnetic beads (MedChem Express, Shanghai, China) that had been pre-conjugated with anti-AP1M1 or anti-AP2M1 (rabbit, Absin, Shanghai, China) and incubated overnight at 4 °C with gentle rocking. For western blotting, cellular proteins were loaded onto a gel and separated via 10% SDS-PAGE, then transferred onto polyvinylidene difluoride (PVDF) membranes (Bio-Rad, Hercules, CA, USA). The PVDF membranes were blocked with 5% (w/v) non-fat milk for 2 h and then incubated with a primary antibody at 4 °C overnight. Anti-Myc, anti-Flag, and anti-β-actin antibodies (Cell Signaling Technology, Beverly, MA, USA) diluted 1:1000 were used to detect the target protein. Following three washes with western washing buffer, the membranes were incubated with the corresponding HRP-conjugated secondary antibody for 1 h at room temperature (Bioss Biotechnology, Beijing, China). The membranes were developed using a Western Lightning chemiluminescence kit (CWBIO, Beijing, China) and imaged by the ChemiDoc MP Imaging System (Bio-Rad, Hercules, CA, USA).

### Indirect immunofluorescence assay

Cells were grown on 24-mm coverslips and transfected with plasmids expressing YLMY motif-mutated F protein and AP1M1–AP5M1. After 24 h, the cells were harvested and fixed in 4% paraformaldehyde in phosphate-buffered saline (PBS) for 15 min at 4 °C. Subsequently, the fixed cells were permeabilized using 0.2% Triton X-100 in 0.1% sodium citrate and blocked with 5% bovine serum albumin. The cells were then incubated with anti-Myc or anti-Flag antibody to detect AP and F protein, respectively, followed by an incubation with a secondary antibody conjugated to Alexa 488 or Alexa 555 (Cell Signaling Technology), and finally counterstained with 4′,6-diamidino-2-phenylindole (DAPI; Sigma) to label the nuclei. Fluorescence signals were captured with a Nikon A1 confocal microscope (Nikon, Tokyo, Japan). The Pearson’s coefficients were calculated by analysing each cell separately in confocal sections.

### Quantification of RNA synthesis by RT-qPCR

NDV-infected BSR-T7/5 cells were collected, and total RNA was extracted using the Cell Total RNA Isolation Kit (Foregene, Chengdu, China). Briefly, the NDV-infected cells were collected at different times for the detection of the virus and cell mRNAs. cDNA was synthesized by reverse transcription with an M5 Super plus qPCR RT kit with gDNA remover (Mei5 Biotechnology, Beijing, China) at 37 °C for 15 min and 85 °C for 5 s, in accordance with the manufacturer’s instructions. The qPCR assays were performed on 20-μl mixtures containing 0.4 μM gene-specific primer, 10 μl of SYBR select master mix, and 2 μl of cDNA template using M5 HiPer SYBR Primeix Estate (Mei5 Biotechnology, Beijing, China) in a LightCycle 96 (Roche, Basel, Switzerland). The PCR parameters were 95 °C for 30 s, followed by 40 cycles at 95 °C for 5 s and 60 °C for 30 s. Gene expression was normalized to that of the housekeeping gene β-actin, and the relative abundance was calculated. Primers were designed using Primer Premier 5.0, and the sequences are as follows: NP-F: 5′-TTACAACTTGGTCGGGGATG-3′, NP-R: 5′-CGATATAAACG CATGAGCTG-3′; F-F: 5′-CTCACTCCTCTTGGCGACTC-3′, F-R: 5′-CTGGTTGGCTTGTATTAGGG-3′; AP1M1-F: 5′-ACCCATGTGAAGCCTTTGAT-3′, AP1M1-R: 5′-CAGGGACCCACT TGACACTC-3′; AP2M1-F: 5′-AAAAGCAGGGCAAAGGCACA-3′, AP2M1-R: 5′-CCAATGGGATCACCCGGAAA-3′; AAK1-F: 5′-GTGAAATGTGCCTTGAAA CG-3′, AAK1-R: 5′-GAATGAGAACTTCCCAGACG-3′; and GAK-F: 5′-CTGGGACC AAACAGCAAGAC-3′, GAK-R: 5′-GCAGCCCAAGAGGAGAAACT-3′.

### Cytotoxicity and drug treatment

The cells were seeded in 96-well plates at a density of 5,000 cells per well and grown overnight. The cells were then treated with inhibitors or small interfering RNA (siRNA) at the indicated concentration for 24 h. The cell viability was tested with CellTiter-Lumi Plus Luminescent Cell Viability Assay Kit (Beyotime Biotechnology, Shanghai, China). The absorbance was measured at 570 nm. The experiments were independently repeated three times. Sunitinib (inhibitor of AAK1) and erlotinib (inhibitor of GAK) were each purchased from MedChemExpress and dissolved in dimethyl sulphoxide (DMSO).

### siRNA transfection

siRNA was transfected into cells using GP-transfect-Mate (GenePharma, Suzhou, China) in accordance with the manufacturer’s protocol 24 h before YLMY-mutant infection. All siRNAs were purchased from GenePharma. Three distinct siRNAs against each gene were tested, and the best one was chosen for use in subsequent experiments. The sequences of the tested siRNAs are as follows: siAP1M1–1, 5′-AAAGUACUCGGAGAAGACCTT-3′; siAP1M1–2, 5′-AACUGCCUCAAUGACG UCCTT-3′; siAP1M1–3, 5′-AUGGACCACACGAUCUCGGCTT-3′; siAP2M1–1, 5′-A UUCGAAGACCAUGGCAGCTT-3′; siAP2M1–2, 5′-UUCGGCGAUACUUGAUGC CTT3′; and siAP2M1–3, 5′AUGGCAAUCGACUGCUUGCTT-3′.

### Membrane protein extraction

F protein and AP expression in the cell membrane structure were analysed using a Membrane and Cytoplasmic Protein Extraction kit (Beyotime Biotechnology) under the specific experimental steps described in the provided protocol. The extracted membrane proteins included not only plasma membrane proteins, but also mitochondria, endoplasmic reticulum, Golgi body membrane, and various vesicle structures. Control experiments were conducted using antibodies against Na/K+ ATPase or β-actin.

### Transmission electron microscopy (TEM)

BSR-T7/5 cells infected with NDV containing a mutated YLMY motif were washed with PBS and centrifuged at 1500 ×g for 5 min. The resulting cell pellets were fixed with 2.5% glutaraldehyde for 24 h at 4 °C and washed with PBS several times. After a post-fixation treatment with 1% osmium tetroxide and 1% potassium ferrocyanide for 1 h at room temperature, the cells were successively transferred through a graded series of ethanol (25%, 50%, 75%, 95%, and 100%) for dehydration. The final pellets were embedded for 48 h in resin at 60 °C for polymerization. Subsequently, ultrathin sections (70 nm) were obtained using the Reichert Ultracut-S ultramicrotome and a diamond knife and then counterstained for 10 min with a 0.5 M aqueous solution of uranyl acetate and lead citrate. Images were observed and recorded using a transmission electron microscope (JEOL, Tokyo, Japan).

### Intra- and extra-cellular virus titre

BSR-T7/5 cells were infected with NDV containing a mutated YLMY motif at a multiplicity of infection of 0.1 at 37 °C for 1 h, then washed three times with PBS and covered with maintenance solution for ongoing culture. At 24 and 36 h post-infection (hpi), the supernatant was collected and centrifuged at 4 °C, 500 ×*g* for 5 min, and the extracellular infective virions were titrated by plaque assay. The remaining cells were washed three times with pre-chilled PBS, digested with 0.25% pancreatin, suspended in maintenance solution, and centrifuged at 1000 ×*g* for 5 min to collect the total cells. The pelleted cells were resuspended with a volume of maintenance solution equal to that of the supernatant, and 100 μl of this cell suspension was freeze-thawed three times (freeze at − 80 °C, thaw at 37 °C), then subjected to centrifugation at 4 °C, 3000 ×*g* for 5 min. The resulting supernatant was collected and used in a plaque assay to determine the intracellular virus titre.

### Data analysis

Variance analysis (ANOVA) methods followed by a Tukey’s test were used to analyse the data. Values presented here are the mean and standard deviation (SD). Differences with a *p*-value of <0.05 were considered as statistically significant. All analyses were performed using Prism 8.0 (GraphPad Software Inc., San Diego, CA, USA).

## Results

### The YLMY motif influences the interaction between NDV F protein and AP complexes

**Figure 1.**
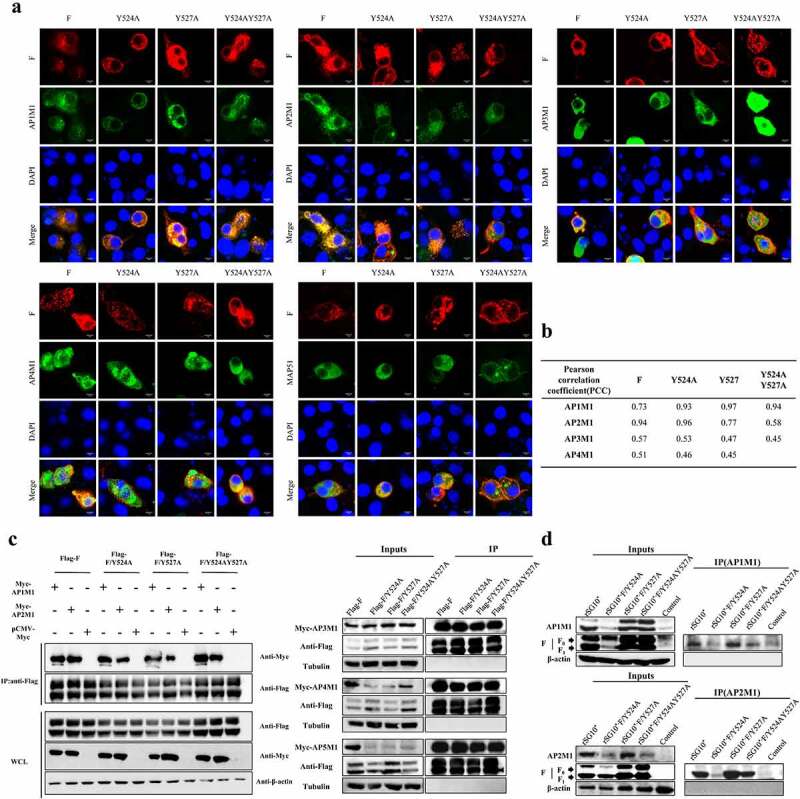
The interaction between NDV F protein and AP complexes following transfection or infection. For *A* and *C*, BSR-T7/5 cells were co-transfected with expression plasmids for an F protein (wildtype, Y524A, Y527A, or YY245 + 527AA) and an AP complex (AP1M1, AP2M1, AP3M1, AP4M1, or AP5M1). (a) at 36 h post-transfection, the cells were fixed, and their nuclei were stained with DAPI. The level of co-localization was observed via laser scanning confocal microscopy. (b) Quantification of the co-localization of F protein and AP under YLMY-motif mutation was performed by calculating the Pearson’s correlation coefficient using ImageJ. (c) Cell lysates were immunoprecipitated (IP) with anti-Flag antibody. The immunoprecipitates and whole-cell lysates (WCL) were analysed by western blotting with anti-Flag, anti-Myc, and anti-Tubulin. (d) BSR-T7/5 cells were infected with NDV containing a wildtype (rSG10*) or mutant (rSG10*-F/Y524A, rSG10*-F/Y527A, or rSG10*-F/Y524AY527A) YLMY motif. At 36 hpi, lysates of these cells were incubated overnight at 4 °C with anti-AP1M1 or anti-AP2M1 pre-conjugated Protein A/G magnetic beads, after which immunoprecipitation was conducted.

Based on our previous finding that the YLMY motif in the CT of NDV F protein regulates its cell surface expression, we presumed that this YLMY motif plays a role in protein transport similar to that of other YXXΦ motifs. Intracellular traffic relies, to a large extent, on the interaction between AP complexes and cargo protein, and this interaction is mediated by recognition of the YXXΦ motif within the cargo protein by subunits of the AP complexes. To identify whether AP complexes interact with NDV F protein, we initially screened the interaction between them using gene expression plasmids; regardless of whether the F protein contained the original YLMY motif or a mutated version, it co-localized with the μ-adaptin for each of five different AP complexes (Figure 1(a,b)). To exclude the possibility of simple co-positioning, we performed co-IP experiments; in agreement with the previous results, all of the F protein versions co-localized with each of the five different AP complexes (Figure 1(c)).

Because AP-1 and AP-2 mediate the formation of clathrin-coated transport vesicles and have the largest cargo repertoires of all AP complex transport vesicles, we selected the μ-adaptins of the AP-1 (AP1M1) and AP-2 (AP2M1) complexes for further study. We validated the interaction between F protein and AP1M1 or AP2M1 in cells infected with an NDV containing a wildtype (rSG10*) or mutated (rSG10*-F/Y524A, rSG10*-F/Y527A, or rSG10*-F/Y524AY527A) YLMY motif. None of the tested YLMY-motif mutations completely impaired the F protein – AP complex interaction, but the quantity of interacting protein varied among the different mutants. Relative to the immunoprecipitation bands for other mutants, the immunoprecipitation band for rSG10*-F/Y524A was weak, and more immunoprecipitation bands could be observed for rSG10*-F/Y527A (Figure 1(d)). Because co-IP experiments are insufficient to prove a direct interaction between proteins, we cannot rule out the possibility that other important proteins in the AP complex may be more directly involved, but the observed difference in immunoprecipitation bands indicates that the YLMY motif indeed regulates the F protein – AP complex interaction.

### AP1M1 and AP2M1 are required for NDV infection

**Figure 2.**
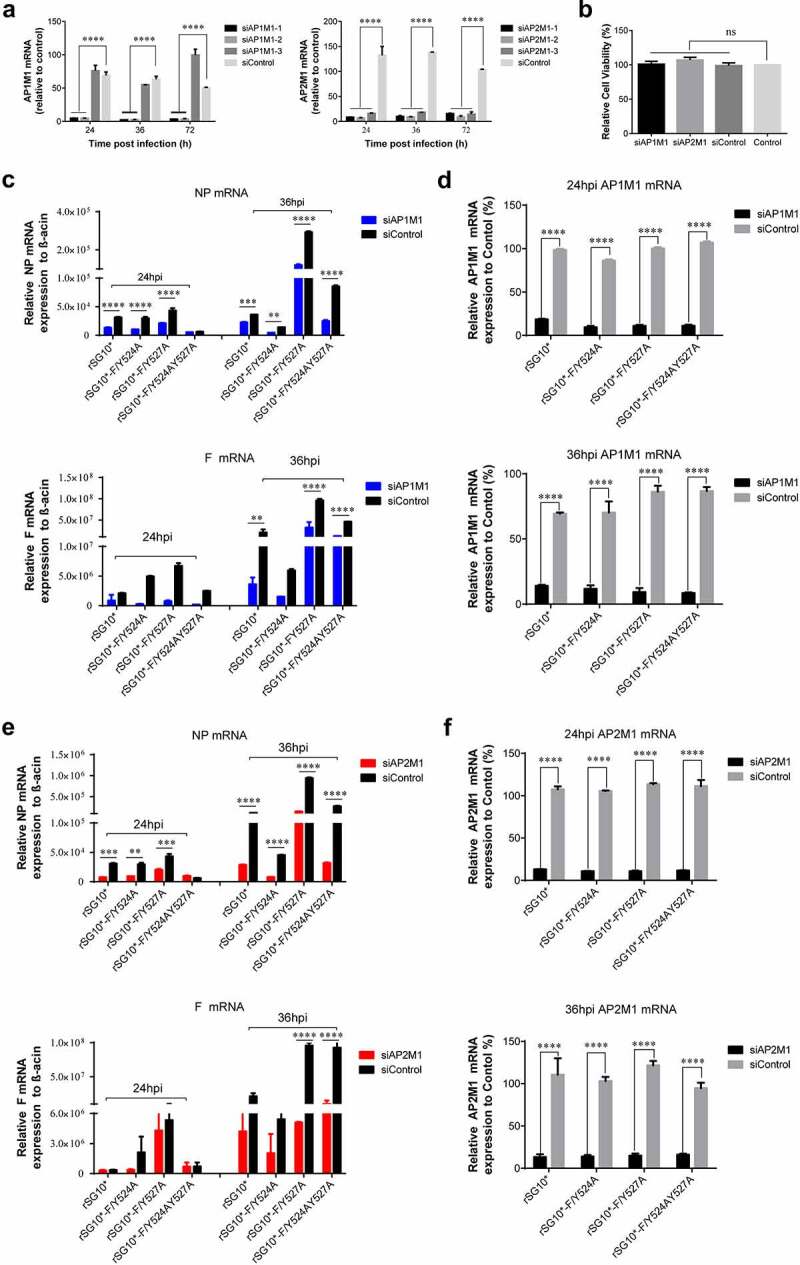
AP1M1 and AP2M1 are required for NDV infection. (a) the effects of different siRNA in the transfected cells were analysed by RT-qPCR. (b) the viability of siRNA-transfected cells. (c) Expression of the NP (top) and F (bottom) genes following infection of AP1M1-knockdown cells with an NDV containing a wildtype (rSG10*) or mutant (rSG10*-F/Y524A, rSG10*-F/Y527A, or rSG10*-F/Y524AY527A) YLMY motif, as measured by qPCR. (d) Expression of AP1M1 in NDV-infected control (siControl-transfected) or AP1M1-knockdown (siAP1M1-transfected) cells. (e) Expression of the NP (top) and F (bottom) genes following infection of AP2M1-knockdown cells with an NDV containing a wildtype (rSG10*) or mutant (rSG10*-F/Y524A, rSG10*-F/Y527A, or rSG10*-F/Y524AY527A) YLMY motif, as measured by qPCR. (f) Expression of AP2M1 in NDV-infected control (siControl-transfected) or AP2M1-knockdown (siAP2M1-transfected) cells. Results shown in panels A – F represent data pooled from at least two independent experiments. The values presented are the mean ± SD. *p <0.05; **p <0.01; ***p <0.001.

To confirm the function of AP1M1 and AP2M1 in NDV infection, siRNA targeting AP1M1 or AP2M1 was transfected into cells before their infection with an NDV strain containing a mutated YLMY motif. The effects of three siAP1M1s and three siAP2M1s were assessed (Figure 2(a)), and siAP1M1–1 and siAP2M1–2 were chosen for use in subsequent experiments; no cytotoxic effects from these siRNAs were observed (Figure 2(b)). qPCR assays revealed that a knockdown of AP1M1 significantly reduced the mRNA levels of NP and F. An analysis of NP and F mRNA revealed that the most pronounced inhibitions to NP and F mRNA levels were in cells infected with rSG10*-F/Y527A (67% and 81%, respectively) or rSG10*-F/Y524AY527A (71% and 74%, respectively), followed by those infected with rSG10* (50% and 66%, respectively), whereas much lower amounts of inhibition of NP and F mRNA levels (37% and 40%, respectively) occurred in cells infected with rSG10*-F/Y524A (Figure 2(c)). AP1M1 expression was consistently suppressed throughout the whole course of NDV infection (Figure 2(d)). A similar pattern of inhibition was observed in the siAP2M1-treated cells (Figure 2(e,f)). The results show that a knockdown of AP1M1 or AP2M1 restrained the level of viral mRNA expression, meaning that AP1M1 and AP2M1 are involved in NDV infection. The inhibition of viral mRNA expression was most obvious for rSG10*-F/Y527A and rSG10*-F/Y524AY527A, whereas it was not as significant for the rSG10*-F/Y524A.

### The NDV YLMY motif regulates the protein expression of AP complexes

**Figure 3.**
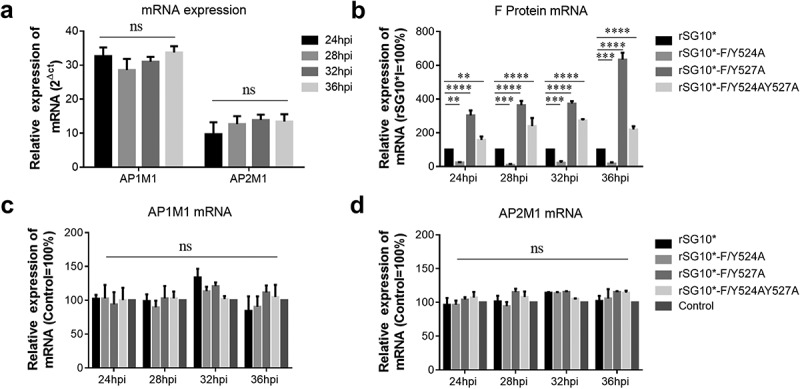
The YLMY motif does not regulate the mRNA expression of AP complexes. (a) the mRNA expression levels of AP1M1 and AP2M1 in control BSR-T7/5 cells at various time points (24, 28, 32, or 36 hpi), as analysed by RT-qPCR. (b–d) the mRNA level of F protein (b), AP1M1 protein (c), or AP2M1 protein (d) in cells infected with an NDV containing a wildtype (rSG10*) or mutant (rSG10*-F/Y524A, rSG10*-F/Y527A, or rSG10*-F/Y524AY527A) YLMY motif. Results in panels a–d represent data pooled from at least two independent experiments. The values presented are the mean ± SD. *p <0.05; **p <0.01; ***p <0.001.

**Figure 4.**
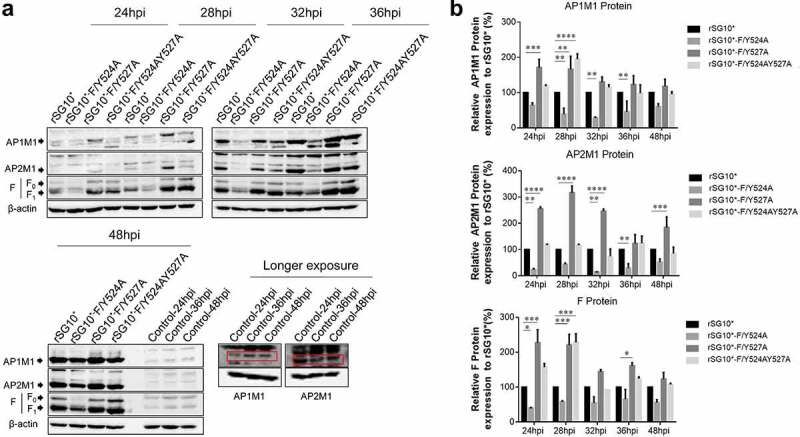
The YLMY motif regulates the protein expression of AP complexes. BSR-T7/5 cells were infected with an NDV containing a wildtype (rSG10*) or mutant (rSG10*-F/Y524A, rSG10*-F/Y527A, or rSG10*-F/Y524AY527A) YLMY motif, at a multiplicity of infection of 0.1. (a) the expression levels of each F protein and host AP1M1 and AP2M1 at various post-infection time points were determined by western blotting. (b) Protein expression levels are expressed as percentages of the levels for rSG10*, which were set at 100%. The *p*-values were calculated by a two-way ANOVA; *p < 0.05; **p <0.01; ***p <0.001; n = 3.

After determining that AP-1 and AP-2 are involved in the NDV lifecycle, we then aimed to determine whether there was a connection between viral F protein and host cell AP by examining the expression of mRNA and protein. We found that the endogenous AP1M1 and AP2M1 mRNA expression levels remained stable between 24 and 36 hpi with NDV (Figure 3(a)). Next, we analysed the viral F protein and host AP mRNA in cells infected with an NDV containing a wildtype or mutant YLMY motif. There was a significant difference in the F protein mRNA levels depending on the YLMY motif status of the infecting virus; compared with rSG10*, rSG10*-F/Y527A and rSG10*-F/Y524AY527A both presented a higher F mRNA level, and the lowest level of F mRNA was exhibited by cells infected with rSG10*-F/Y524A (Figure 3(b)). In contrast, the mRNA levels of AP1M1 and AP2M1 did not differ between the NDV mutants (Figure 3(c,d)). We then compared the protein expression levels of AP1M1, AP2M1, and F protein and found that AP1M1 and AP2M1 expression were upregulated following NDV infection, regardless of whether the YLMY motif contained a mutation, compared with the levels in uninfected cells, in which weak bands could be detected only after extending the exposure time (Figure 4(a)). The degree of upregulation was different among the cells infected with the various YLMY motif mutants; the expression levels of AP1M1 and AP2M1 were particularly high in cells infected with rSG10*-F/Y527A or rSG10*-F/Y524AY527A, whereas they were only slightly higher than the control cell levels in cells infected with rSG10*-F/Y524A. The trends of AP1M1 and AP2M1 protein expression are consistent with those of the viral F protein (Figure 4(b)). These data suggest that YLMY motifs regulate the protein expression but not the mRNA expression of host cell AP and that the difference in F protein expression may be related to the protein level of AP.

### The YLMY motif is important for F protein transport by AP-1 and AP-2

**Figure 5.**
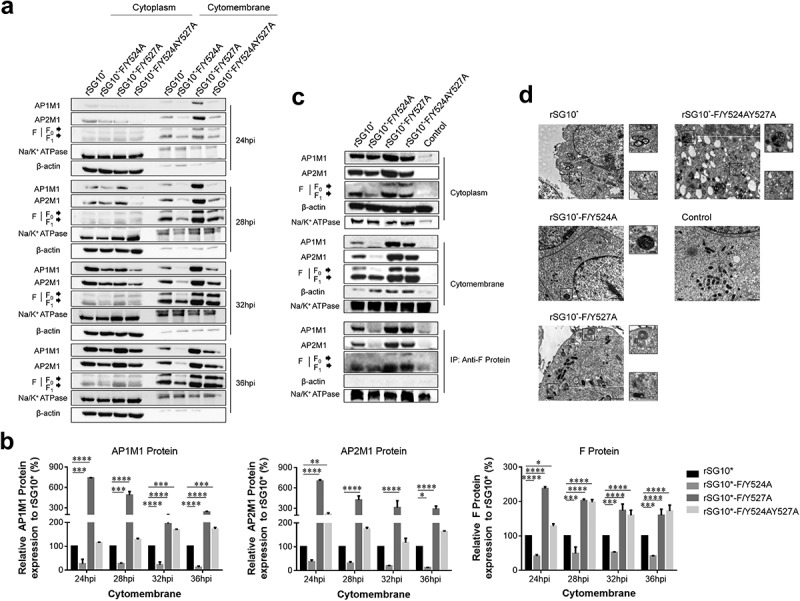
The viral F protein is transported by AP-1 and AP-2. (a) Expression of AP1M1 and AP2M1 in the membrane protein fraction was determined by western blot. BSR-T7/5 cells were infected with an NDV containing a wildtype (rSG10*) or mutant (rSG10*-F/Y524A, rSG10*-F/Y527A, or rSG10*-F/Y524AY527A) YLMY motif, at a multiplicity of infection of 0.1. The membrane protein was extracted, following the kit instructions, at different post-infection time points. (b) Quantitative analysis of AP1M1, AP2M1, and viral F protein expression levels, expressed as percentages of the levels for rSG10* (set at 100%). (c) a co-IP conducted on the membrane proteins. After cells were infected with an NDV containing a wildtype or mutant YLMY motif, the membrane protein fraction was extracted and used in an immunoprecipitation performed with an anti-F antibody. (d) TEM micrographs of infected cells at 36 hpi. White arrows mark the presence of many lysosomes and autophagosomes, and black arrows indicate transport vesicles in the cytoplasm. N, nucleus; ER, endoplasmic reticulum.

NDV F protein is transported to the host cell surface where it participates in viral budding, and we found that NDV strains containing YLMY motifs with different mutated positions exhibited differences in this process. An AP-composed vesicle can mediate cargo protein traffic, and our previous experiment described above demonstrated that NDV F protein interacts with AP. Given the consistent expression of F protein and AP, we speculated that AP is associated with the process of F protein trafficking to the cell membrane. We extracted membrane proteins from NDV-infected cells and found that the intracellular expression levels of host AP and the F protein from NDVs with a mutated YLMY motif were similar, but their membrane expression levels were quite different (Figure 5(a)). A cytomembrane protein analysis revealed that the host AP1M1 and AP2M1 expression levels in cells infected with rSG10*-F/Y527A or rSG10*-F/Y524AY527A were higher than those in cells infected with rSG10*, especially in the early stages of viral infection; in contrast, the host AP1M1 and AP2M1 expression levels in cells infected with rSG10*-F/Y524A were lower than those in cells infected with rSG10* throughout the whole infection. The same trend seen for AP1M1 and AP2M1 expression levels was also observed for viral F protein (Figure 5(b)).

To confirm that these matching trends in expression were caused by AP transporting F protein to the cell surface, a co-IP was conducted with membrane fractions derived from NDV-infected cells. We observed the expected interaction between F protein and AP in the membrane fraction (Figure 5(c)). AP-1 and AP-2 mediate the formation of transport vesicles and recognize cargo proteins via typical sorting motifs. The results of our co-IP experiment demonstrated that NDV F protein interacts with AP complexes, and the trends in AP1M1 and AP2M1 protein expression were consistent with those of YLMY-mutant F proteins, especially in the membrane protein, which contains vesicles composed of APs. Together, these results indicate that the YLYM motif affects AP-1 and AP-2 mediation of NDV F protein trafficking. In support of this conclusion, our electron microscopy results show that many transport vesicles containing light particles were abundant in cells infected with rSG10*-F/Y527A or rSG10*-F/Y524AY527A but such vesicles were extremely rare in cells infected with rSG10*-F/Y524A. Additionally, vesicles resembling lysosomes or autophagosomes, some of which were in close vicinity to vesicular packets, were commonly observed in cells infected with rSG10*-F/Y527A or rSG10*-F/Y524AY527A, whereas such vesicles were observed only rarely in cells infected with rSG10*-F/Y524A (Figure 5(d)).

**Figure 6.**
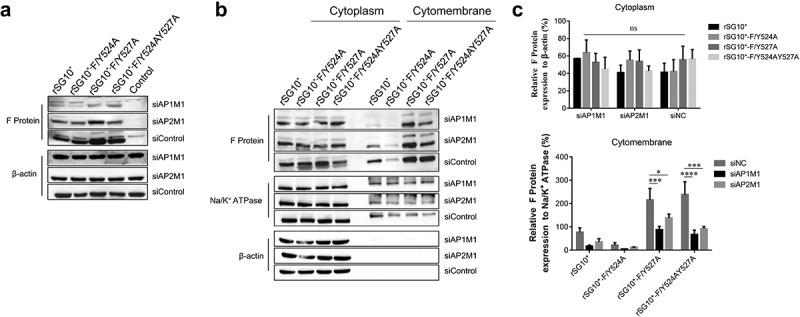
Treatment with siAP1M1 or siAP2M1 impairs the expression and transportation of mutant F protein. (a) at 24 h after cells were transfected with siAP1M1 or siAP2M1, they were infected with an NDV containing a wildtype (rSG10*) or mutant (rSG10*-F/Y524A, rSG10*-F/Y527A, or rSG10*-F/Y524AY527A) YLMY motif. The F protein expression level in these cells was measured by western blot. (b) the proteins of cells treated as above were separated into membrane and cytoplasm fractions before being subjected to a western blot. (c) Quantitative analysis of the viral F protein expression from part (b). Protein expression levels are presented as percentages of the β-actin expression level. The *p*-values were calculated by a two-way ANOVA; *p <0.05; **p <0.01; ***p <0.001; n = 3.

To determine if AP has a direct effect on NDV F protein transportation, we transfected cells with siAP1M1 or siAP2M1 before infecting them with NDV and analysed the effects of these siRNAs on F protein expression. The F protein expression was reduced by siRNA treatment, especially in cells infected with rSG10*-F/Y527A or rSG10*-F/Y524AY527A (Figure 6(a)). Treatment with siAP1M1 or siAP2M1 minimally affected the F protein expression in the cytoplasm but more drastically altered its expression in the membrane (Figure 6(b)). Compared with the control siRNA-treated cells, the cells treated with siAP1M1 or siAP2M1 had lower levels of F protein expression detected in the membrane fraction when they were infected by rSG10*-F/Y527A or rSG10*-F/Y524AY527A but not when they were infected by rSG10*-F/Y524A (Figure 6(c)). Collectively, these results provide evidence that the similarity between the protein expression trends of AP and F protein is associated with AP-mediated F protein trafficking and that the YLMY motif affects this process.

### AP mediates the viral titres of NDVs expressing a mutated YLMY motif

**Figure 7.**
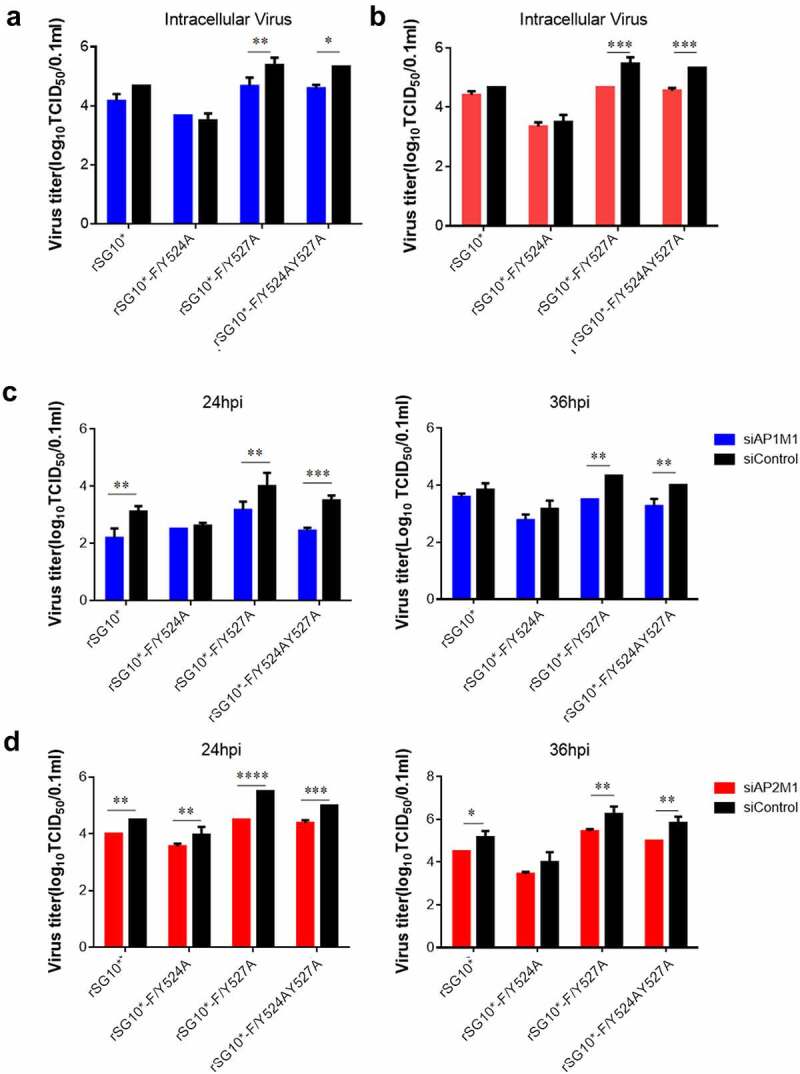
AP regulates the intracellular and supernatant viral titres of NDVs with a mutated YLMY motif. The viral infectivity was measured as the tissue culture infective dose by inoculating naive cells with lysates (intracellular virus) or supernatants (extracellular virus) from NDV-infected cells in which AP1M1 or AP2M1 had been depleted via siRNA treatment. (a–d) the intracellular (a and b) or extracellular (c and d) virus titer of siAP1M1-treated (a and c) or siAP2M1-treated (b and d) cells infected with an NDV containing a wildtype (rSG10*) or mutated (rSG10*-F/Y524A, rSG10*-F/Y527A, or rSG10*-F/Y524AY527A) YLMY motif.

After finding that AP-1 and AP-2 are related to NDV F protein transportation, we then assessed the influence of siAP1M1 and siAP2M1 on virus titres. The knockdown of AP1M1 or AP2M1 significantly reduced the intracellular viral titres of rSG10*-F/Y527A and rSG10*-F/Y524AY527A but did not affect the intracellular viral titer of rSG10*-F/Y524A (Figure 7(a,b)). We then analysed the extracellular viral titers in cells treated with siAP1M1 or siAP2M1 and noticed that the knockdown of AP1M1 or AP2M1 also reduced the extracellular viral titer of NDV, especially for rSG10*-F/Y527A and rSG10*-F/Y524AY527A, but not for rSG10*-F/Y524A (Figure 7(c,d)). Vesicles composed of AP mediate the transport to the cell membrane of viral F protein, where this viral protein is subsequently involved in viral assembly and budding and the release of infectious virions. The influence of an AP knockdown on the NDV titer suggests that AP regulates F protein transport to the cell surface, which in turn affects the infectious viral titer.

### The YLMY motif influences the expression of p-AP2M1, AAK1, and GAK in NDV-infected cells

**Figure 8.**
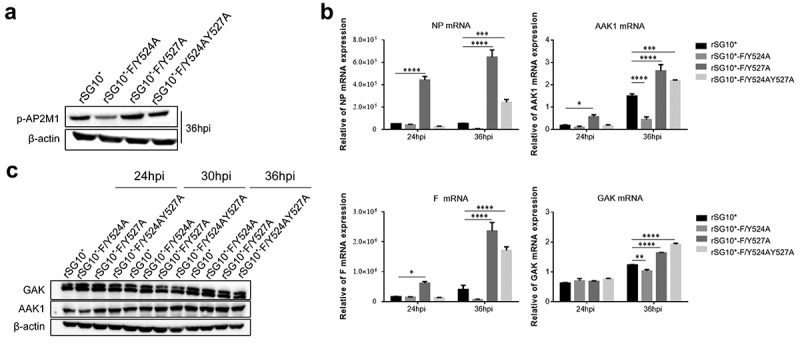
The expression of *p*-AP2M1, AAK1, and GAK in cells infected with an NDV containing a wildtype or mutated YLMY motif. Cells were infected with an NDV containing a wildtype (rSG10*) or mutated (rSG10*-F/Y524A, rSG10*-F/Y527A, or rSG10*-F/Y524AY527A) YLMY motif. (a) the phosphorylation of AP2M1 in these cells was detected by western blot. (b) the mRNA expression levels of viral NP and F genes and host AAK1 and GAK genes in these cells were analysed by RT-qPCR. The *p*-values were calculated by a two-way ANOVA; *p <0.05; **p <0.01; ***p <0.001; n = 3. (c) Protein expression of host AAK1 and GAK in these cells.

The experiments described above revealed that the NDVs containing a mutated YLMY motif have defects in AP targeting and F protein translocation. Given the known mechanism by which AP transports cargo protein, we first assessed the phosphorylation of AP2M1. The *p*-AP2M1 expression was found to be quite different in cells infected with an NDV containing a mutated YLMY motif, which is consistent with our findings for AP and F protein (Figure 8(a)). Thus, the viruses with a mutated YLMY motif differ from those with a wildtype YLMY motif in their regulation of AP. We then analysed the mRNA and protein expression levels of AAK1 and GAK; the mRNA levels of AAK1 and GAK were differently regulated by the YLMY motif mutants, and their variation trends were similar to those for viral NP and F mRNA. Compared with rSG10*, rSG10*-F/Y527A and rSG10*-F/Y524AY527A, which have higher NP and F mRNA levels, were also found to have higher mRNA levels of AAK1 and GAK. Additionally, for rSG10*-F/Y524A, which presented the lowest expression levels of NP and F, the AAK1 and GAK mRNA levels were also the lowest (Figure 8(b)); however, this phenomenon was not observed for the protein expression levels of AAK1 and GAK (Figure 8(c)). One possible explanation for the different influence of the YLMY motif on AAK1 and GAK mRNA and protein expression may be that its influence on mRNA expression is not strong enough to be reflected at the protein level. Together, these results suggest that the YLMY motif influences, to some extent, the expression of *p*-AP2M1, AAK1, and GAK.

### AAK1 and GAK inhibitors have different effects on mRNA and protein expression in cells infected with NDV containing a mutated YLMY motif

**Figure 9.**
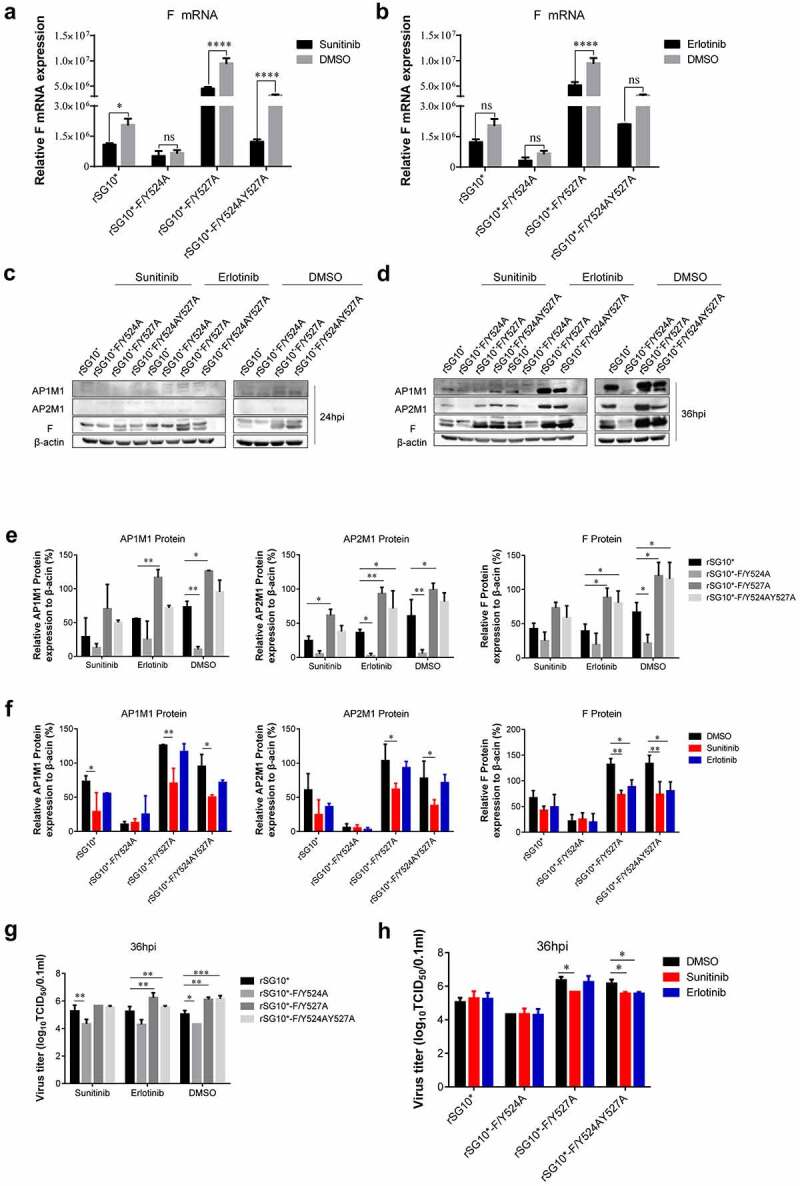
The influence of inhibitors sunitinib and erlotinib on NDV F, AP1M1, and AP2M1 protein expression. (a and b) Viral F mRNA expression of cells infected with an NDV containing a wildtype (rSG10*) or mutated (rSG10*-F/Y524A, rSG10*-F/Y527A, or rSG10*-F/Y524AY527A) YLMY motif and treated with sunitinib (a) or erlotinib (b). The cells were infected with NDV at 37 ℃ for 1 h, and then the cells were treated with 1 µm sunitinib or 10 µm erlotinib; DMSO was used as a negative control. The *p*-values were calculated by a two-way ANOVA; *p <0.05; **p <0.01; ***p <0.001; n = 3. (c and d) the F, AP1M1, and AP2M1 protein expression at 24 hpi (c) or 36 hpi (d) in cells infected with an NDV containing a wildtype (rSG10*) or mutated (rSG10*-F/Y524A, rSG10*-F/Y527A, or rSG10*-F/Y524AY527A) YLMY motif and treated with sunitinib or erlotinib. (e) Quantitative analysis of the viral F and host AP1M1 and AP2M1 protein expression by treatment group (sunitinib, erlotinib, or DMSO). (f) Quantitative analysis of the viral F and host AP1M1 and AP2M1 protein expression by NDV strain (rSG10*, rSG10*-F/Y524A, rSG10*-F/Y527A, or rSG10*-F/Y524AY527A). (g) Virus titre of each treatment group (sunitinib, erlotinib, or DMSO). The virus titer was measured as the tissue culture infective dose by inoculating naive cells with supernatants from infected cells. (h) Virus titer of each NDV strain (rSG10*, rSG10*-F/Y524A, rSG10*-F/Y527A, or rSG10*-F/Y524AY527A). The *p*-values were calculated by a two-way ANOVA; *p <0.05; **p <0.01; ***p <0.001; n = 3.

AAK1 and GAK are protein kinases, so we used the inhibitors sunitinib (targeting AAK1) and erlotinib (targeting GAK) to investigate the differences in AP recognition and F protein transportation among the NDVs containing mutated YLMY motifs. We first performed cytotoxicity tests to determine the effects of various drug concentrations on cell viability. From the results, 1 µM sunitinib and 10 µM erlotinib were selected for use as our working concentrations (Supplementary Figure S1(A) and (B)). Sunitinib treatment of cells infected with an NDV containing a mutated YLMY motif reduced the F mRNA expression, especially in cells infected with rSG10*-F/Y527A or rSG10*-F/Y524AY527A, but this effect was not observed in cells infected with rSG10*-F/Y524A (Figure 9(a)). Erlotinib treatment also suppressed the F mRNA expression in NDV-infected cells, but this inhibitory effect was significant only for cells infected with rSG10*-F/Y527A (Figure 9(b)). This suggests that inhibiting AAK1 activity had a more pronounced effect on the YLMY mutants.

We then tested the effect of these inhibitors on protein expression. After adding sunitinib or erlotinib to NDV-infected cells, no noticeable differences in the protein expression levels of F protein, AP1M1, or AP2M1 were observed at 24 hpi (Figure 9(c)). However, at 36 hpi, the levels of detected protein expression were all lower in the inhibitor-treated groups than in the DMSO group, especially the sunitinib (AAK1 inhibitor) groups (Figure 9(d)). A quantitative analysis of the expressed protein indicated that inhibitor treatment did not completely change the expression trends of F protein, AP1M1, and AP2M1 between each mutant, but the gap in expression levels among them was narrowed (Figure 9(e)). Compared with the other groups, the effects of inhibition by sunitinib on F, AP1M1, and AP2M1 protein expression were most obvious in the rSG10*-F/Y527A and rSG10*-F/Y524AY527A groups (Figure 9(f)).

Finally, we measured the viral titre after inhibitor treatment. Consistent with the protein expression findings, the viral titer of each NDV with a mutated YLMY motif was decreased to some extent by inhibitor treatment, especially in the rSG10*-F/Y527A and rSG10*-F/Y524AY527A groups (Figure 9(g,h)). Together, these results imply that inhibiting the activity of AAK1 or GAK suppresses NDV viral replication and protein expression, especially for rSG10*-F/Y527A and rSG10*-F/Y524AY527A. The NDVs with mutated YLMY motifs exhibit some differences in their levels of µ1 and µ2 phosphorylation by AAK1 and/or GAK kinase, enabling conformational change and cargo protein-binding of AP.

## Discussion

Envelope glycoproteins are key components of viral particles; they are essential for efficient viral infectivity because they bind the viral receptor on host cells and mediate membrane fusion. Consequently, these glycoproteins are the main target for both host antibody responses and vaccine development [43]. For viral propagation, it is essential that glycoproteins are precisely transported to the virus assembly and budding site. During their co-evolution with host organisms, viruses have acquired genome sequences that are identical or very similar to the motifs that host proteins use to interact with AP complexes. Although some research on intracellular viral glycoproteins has been conducted [44,45], data on the targeting of NDV F protein in NDV-infected cells are limited. Previous research revealed that the mediation of viral pathogenicity by the YLMY motif depends mainly on regulating transportation of F protein to the cell surface. It was unknown which cellular adaptors are essential for F protein trafficking and the mechanism by which the YLMY motif is involved. In the present study, we set out to address this knowledge gap. Based on our previous research, we hypothesized that the YLMY motif, functioning as a YXXΦ motif, is related to AP-mediated F protein transportation.

Confocal microscopy and co-IP results revealed the co-localization of mutated-YLMY-motif F proteins and AP complexes in cells transfected with expression plasmids. Because AP-1 and AP-2 have the largest cargo repertoires among the AP complex transport vesicles [34,46] and have been demonstrated to be involved in host infection by other viruses [47,48], we decided to investigate the interaction between AP-1 or AP-2 and F protein in cells infected with NDVs containing mutated YLMY motifs. All tested viruses interacted with both AP-1 and AP-2, but the amount of the interacting proteins varied (Figure 1). We propose two plausible explanations for the observed variation in protein interacting levels: 1) the position of the YLMY motif mutation might affect the recognition of F protein by AP, resulting in varied expression levels of each protein; or 2) mutations to the YLMY motif might affect only the expression of the viral F protein. The 527 tyrosine mutation had a stronger effect compared with the 524 tyrosine mutation, resulting in the 524 + 527 dual mutation having the same effect on viral protein and host AP expression as the 527 tyrosine mutation. This phenomenon was reported for varicella-zoster virus (VZV), in which the mutation of ORF9p leucine 231 results in a complete loss of the interaction between ORF9p and AP-1, as evidenced by the lack of co-localization, suggesting that ^227^EGLNLI^232^ is critical to this interaction. Because the YLMY motif 524 and 527 single-point mutations and combined mutations did not eliminate the interaction of F protein with AP completely, we also aimed to investigate whether the YLMY motif functions in a holistic way. We attempted to delete the YLMY motif and rescue a mutant strain without success. The rescue failure could be ascribed to the lethal effect of a YLMY motif deletion. Therefore, we conducted follow-up research only on the point mutant strain.

To confirm the participation of AP in NDV infection, siAP1M1 and siAP2M1 were applied. The qPCR assays revealed that a knockdown of AP1M1 or AP2M1 reduced the mRNA levels of NP and F in all mutant and parental strain viruses, indicating that AP1M1 and AP2M1 are both involved in NDV infection. The viral mRNA inhibition by the AP1M1 or AP2M1 knockdown was most obvious in cells infected with rSG10*-F/Y527A (67% for NP and 81% for F) or rSG10*-F/Y524AY527A (71% for NP and 74% for F), with progressively less inhibition in cells infected with rSG10* (50% for NP and 66% for F) or rSG10*-F/Y524A (37% for NP and 40% for F) (Figure 2). The difference in the reduction of mRNA levels may be due to differences in the utilization of AP complexes by different mutant strains. AP complexes have been widely proven to participate in different stages of infection by other viruses in the same family as NDV [41,46]; for example, for other Paramyxoviridae family members NiV and HeV, only AP-1 and AP-3, respectively, have been proven to be involved in their lifecycles [49,50]. Our data indicate that APs are also essential for NDV infection and reveal that the YLMY motif mediates this process.

The diverse AP expression, observed via interaction assays, in the cells infected by NDVs with mutated YLMY motifs suggests that the YLMY motif probably mediates AP expression. We first tested the mRNA levels of host AP and viral genes and noticed that the expression level trends were not similar between AP and viral genes (Figure 3). We subsequently analysed the correlation with their protein expression. Surprisingly, the protein expression level of AP in NDV-infected cells was clearly higher than that in control cells, indicating that all the NDVs containing a mutated YLMY motif upregulated the protein expression of host cell AP. Furthermore, the protein expression trend for AP was consistent with that for F protein in cells infected with the various YLMY-motif-mutant NDVs (Figure 4). We did not observe a consistent trend at the mRNA level, indicating that the effect of the YLMY motif on the protein expression of AP was not achieved by regulating the mRNA process; the further mechanism may be related to key protein and kinase activities, protein transport, and accumulation. Substantial evidence suggests that many viral genera use cellular AP complexes for their own benefit, sometimes by hijacking mechanisms that allow cargo to be transported to appropriate locations [49,50].

The above results suggest that NDV may exploit host AP complexes to transport F protein, and a previous analysis concluded that the YLMY motif regulates NDV replication and pathogenicity by affecting the transport of F protein to the cell surface [42]; from these findings, we inferred that the YLMY motif mediates the process of F protein transportation by AP to the cell surface. The results of our membrane protein assay show that mutant F proteins exhibited expression trends in the membrane fraction that are like those of host AP in the membrane fraction, and these proteins interact with each other. Additionally, TEM analyses revealed greater numbers of transport vesicles in cells infected with mutated-YLMY-motif NDVs (Figure 5). To specifically investigate the effect of AP on F protein trafficking, siRNA was used. The whole cellular expression level of F protein, but particularly the membrane component, was significantly reduced by a knockdown of AP1M1 or AP2M1, especially in cells infected with rSG10*-F/Y527A or rSG10*-F/Y524AY527A (Figure 6). These findings support our hypothesis that NDV uses its YLMY motif to hijack the host AP system for the transport of viral F protein. We then analysed the intracellular and extracellular viral titres of AP1M1- or AP2M1-depleted cells that were infected with mutated-YLMY-motif NDVs. In the rSG10*-F/Y527A and rSG10*-F/Y524AY527A groups, the knockdown of AP complexes caused a statistically significant decrease in the intracellular virus titer. The extracellular viral titres of each mutant virus were also decreased, to some extent, by AP complex knockdown. These results suggest that the depletion of AP inhibits F protein transportation to the cell surface, thereby leading to a defect in viral titer; however, this inhibition of viral titer by AP depletion is not pronounced for rSG10*-F/Y524A. In other paramyxoviruses, the M proteins of both NiV and HeV are bound by the β3-adaptin of AP-3, and inhibition of these interactions reduces viral particle production, suggesting that the M protein hijacks the host cellular trafficking pathway [51]. For NDV M protein, a mutation of its nuclear localization signal attenuated viral replication and pathogenicity, in a manner associated with host cell protein transcription, processing, and transport [52]. In the case of HIV-1, the role of AP complexes in the assembly and release of enveloped virus is already well established. Specifically, HIV-1 Gag protein interacts with the δ-subunit of AP-3, and a loss of AP3 impaired the assembly and release of HIV-1 particles [53].

Phosphorylation of the AP-2 μ subunit by AAK1 and GAK enhances their binding to sorting motifs within the cargo. Previous results suggested that the YLMY motif mediates the transportation of F protein by AP, and our AP2M1 phosphorylation findings are in agreement with this conclusion (Figure 8(a)). We then focused on determining the specific mechanism of this process. Although the trends in mRNA expression levels for host kinases AAK1 and GAK are similar to those for the viral NP and F genes in cells infected with mutated-YLMY-motif NDVs, we did not observe this phenomenon at the protein level (Figure 8). Presumably, we examined the levels of overall AAK1 and GAK protein expression, rather than the levels of only their activated forms. Using pharmacological inhibitors of AAK1 and GAK, we further established that these kinases influence the replication and protein expression of mutated-YLMY-motif NDVs. Treatment with these inhibitors suppressed viral gene replication, especially for cells infected with rSG10*-F/Y527A or rSG10*-F/Y524AY527A that were then treated with sunitinib, but this inhibition was not so pronounced for cells infected with rSG10*-F/Y524A. Treatment with sunitinib or erlotinib did not entirely change the trends in expression levels of viral F protein and host AP1M1 and AP2M1 in cells infected with mutated-YLMY-motif NDVs, but it bridged the gap in expression levels between the mutants. The inhibition of protein expression was more noticeable in cells infected with rSG10*-F/Y527A or rSG10*-F/Y524AY527A, and we noticed the same inhibition trend regarding viral titre (Figure 9). Together, these results strongly indicate that the NDVs with different YLMY motif mutants differ in their processes of F protein transport by AP; when inhibiting this process with drugs, the suppression of viral replication and protein expression was more obvious for rSG10*-F/Y527A and rSG10*-F/Y524AY527A. GAK activates EPN1 and regulates the uncoating of clathrin-coated vesicles (CCVs) [54], allowing the clathrin to be recycled back to the cell surface, while AAK1 phosphorylation of NUMB [55] mediates the quick recycling of receptors from early/sorting endosomes back to the cell membrane [56]. Compared with the effects of inhibition by erlotinib (targeting GAK) on the cells infected with mutated-YLMY-motif NDVs, those of inhibition by sunitinib (targeting AAK1) were more obvious. The distinction between these two drugs will be useful for further research.

Viruses are exclusively intracellular pathogens that depend on host cellular mechanisms at different stages of their lifecycle. Precise localization of viral protein is required to guarantee the production of progeny viruses. Our study found that host AP interacts with NDV F protein and transports it to the cell surface and that the YLMY motif located in the F protein CT mediates this process. Identifying and targeting universal host trafficking pathways used by irrelevant viral pathogens is a promising approach to advance the discovery of new antiviral therapeutics. A recent study showed that *N*-(p-amylcinnamoyl) anthranilic acid could interrupt the AP2M1–virus interaction and exhibited in vivo activity against multiple viruses, including influenza A virus, Zika virus, Middle East respiratory syndrome-related virus, and severe acute respiratory syndrome coronavirus [50]. Analyzing the virus – AP interaction is a prerequisite for gaining a better understanding of both currently recognized and undiscovered host targets that are hijacked by multiple virus families. In the present work, we constructed and rescued mutant viruses based on the genotype Ⅶ virulent strain SG10, and our results reveal the key influence of the YLMY motif on the biological characteristics of NDV and on the interaction between viral and host proteins. Notably, the findings of the current study do not support those of previous research, which found that the substitution of single tyrosine residues in intermediate virulent strains did not reduce the expression of F protein on the cell surfaces [57]. This inconsistency suggests that although the YLMY motif is highly conserved in various genotypes of NDV, there is a complex regulatory mechanism for strains of differing virulence. Therefore, the effect of this motif on an avirulent strain should be investigated in a follow-up study.

In conclusion, in this study we found that NDV F protein interacts with AP, which is involved in the life cycle of NDV and mediates the transport of F protein to the cell surfaces. Mutations in the YLMY motif affect the interaction, thereby regulating host AP-mediated F protein transport.

## Supplementary Material

Supplemental Material

## Data Availability

The authors confirm that the data supporting the findings of this study are available within the article.
