# Supplementary figures and images for: Adaptor complex-mediated trafficking of Newcastle disease virus fusion protein is regulated by the YLMY motif of its cytoplasmic tail
Source: Virulence. 2022 Oct 18;13(1):1849–67. doi: 10.1080/21505594.2022.2136433 (PMC9586687; doi:10.1080/21505594.2022.2136433)

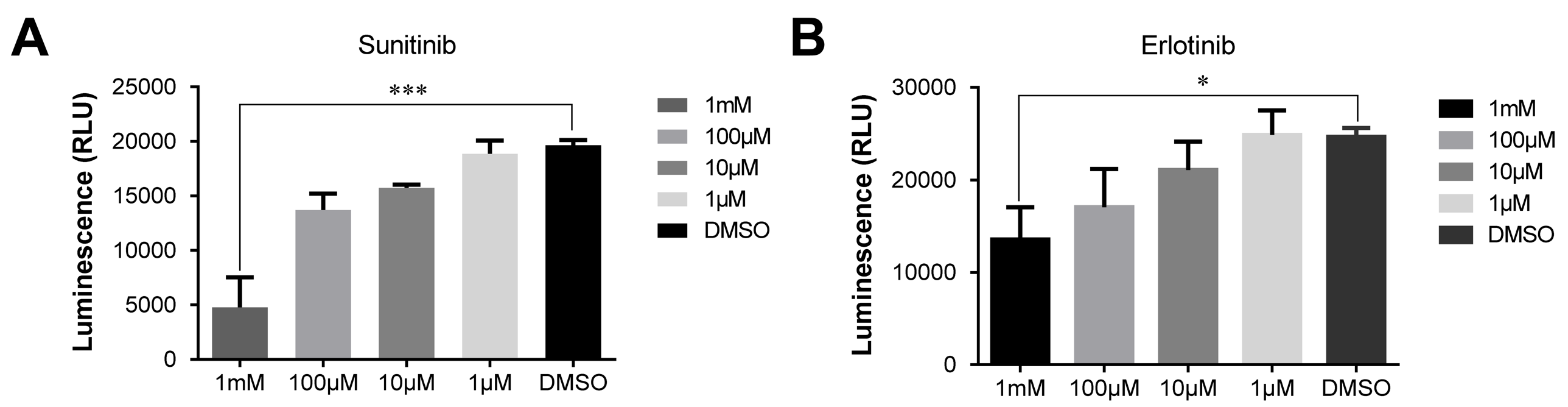

Supplement: Supplemental Material [file KVIR_A_2136433_SM2462.tif]
